# Correlates of prevalent syphilis infection among men who have sex with men (MSM) living with HIV attending the HIV clinic in Trinidad

**DOI:** 10.1371/journal.pone.0265909

**Published:** 2022-03-31

**Authors:** R. Jeffrey Edwards, Abigail Mohammed, Leon-Omari Lavia, Jonathan Edwards, Shiva Verma, Saketh Reddy, Gregory Boyce

**Affiliations:** 1 Medical Research Foundation of Trinidad and Tobago, Port of Spain, Trinidad; 2 University of the West Indies, Department of Paraclinical Sciences, St Augustine, Trinidad; International AIDS Vaccine Initiative, UNITED STATES

## Abstract

**Background:**

Syphilis among men who have sex with men (MSM) living with HIV is of public health concern, thus the objective of the study is to determine the correlates of prevalent syphilis infection in this population so that urgent interventions could be instituted.

**Methods:**

A cross-sectional study was conducted during the period September 2020-June 2021 among MSM who attended a large HIV Clinic in Trinidad. A questionnaire was administered to obtain socio-demographic data and correlates of prevalent syphilis and patients were screened for syphilis using the traditional algorithm. Descriptive and bivariate analyses were conducted and multivariable logistic regression factors was used to assess factors significantly associated with a syphilis diagnosis.

**Results:**

Two hundred and sixty four MSM were enrolled; age range 18–64 years, median age 33 years and 89 (34.4%) were actively bisexual. The prevalence of syphilis was 28% (74/264) and 89.2% (66/74) of these infections were asymptomatic. Multivariable logistic regression analysis showed that those patients who participated in sex with anonymous partners (OR 2.09; 95% CI, 1.03–4.26), those with a previous diagnosis of syphilis (OR 5.16; 95% CI, 1.03–25.83) and those who used marijuana in the last 12 months (OR 2.13; 95% CI, 1.14–3.96) were more likely to be diagnosed with syphilis.

**Conclusion:**

There is a high prevalence of asymptomatic syphilis among MSM living with HIV in Trinidad. Repeat episodes of syphilis and anonymous sex may play a role in the transmission dynamics of *T pallidum* infection in this population, thus urgent public health prevention interventions are warranted.

## Introduction

Syphilis is a sexually transmitted infection (STI) caused by the gram negative spirochete, *Treponema pallidum*, which has been linked to increased sexual transmission of HIV [1] and disproportionately affects men who have sex with men (MSM) [2]. Tsuboi et al [3] conducted a global systematic review and meta-analysis of the prevalence of syphilis among MSM over the period 2000–2020 and found the global pooled prevalence was unacceptably high at 7.5%, and ranged from 1.9% in Australia and New Zealand to 10.6% in Latin America and the Caribbean [3].

The twin island republic of Trinidad and Tobago (T&T) are the southernmost islands of the Caribbean with an estimated population of 1.39 million persons (2020 mid-year estimate) and an ethnic composition comprising 35.4% of persons of East Indian descent, 34.2% of persons of African origin, 23.0% of mixed races, and 8.4% of other ethnic groups (European, Asian, Middle Eastern). The first cases of AIDS in T&T were reported in MSM in 1983 [4], but by 1985 there was an evolution to predominantly heterosexual transmission of HIV [5]. Currently T&T has approximately 10,000 persons living with HIV (PLHIV) with an adult prevalence of 0.7% [6] and the HIV prevalence among MSM in 2015 was 26.6% [7]. All PLHIV in T&T benefit from a national policy of subsidized HIV care and treatment, “treat all” was instituted in 2017 whereby all PLHIV are offered antiretroviral therapy (ART) regardless of CD4 count [8] and this has contributed in part to the number of incident cases of HIV in T&T in 2020 being less than 200.

The prevalence of STIs among PLHIV attending the STI Clinic in Trinidad was found to be 35.1% in 2012 [9] and 37.1% in 2014 [10] in two cross-sectional studies and STIs were found to be more common in MSM in whom syphilis was the most commonly diagnosed STI [10]. In these studies, the prevalence of syphilis increased from 13.2% in 2012 (9) to 21.0% in 2014 [10]. A retrospective study was conducted in 2019 among MSM living with HIV attending the HIV clinic in Trinidad [11] and showed the prevalence of syphilis was 41.3%, most of these infections were asymptomatic (71.1%) and syphilis was more likely to be diagnosed among patients in the 30–34 year old age group and those who were previously treated for syphilis [11]. Stigma and discrimination still persist in T&T as relationships among members of the same sex are not readily accepted by the general population and by some health care workers [11], though in 2018, anal sex among consenting adults was decriminalized [12].

The high prevalence of syphilis among MSM living with HIV is a cause for concern, thus the main objective of the present study is to determine the correlates of prevalent syphilis infection in this population so that urgent public health interventions could be instituted.

## Subjects and methods

The Medical Research Foundation of Trinidad and Tobago (MRFTT) is the largest HIV Treatment and Care Centre in the Trinidad and Tobago where daily clinics are held via appointments and walk-in visits. As of September 2020, there were 6,833 patients currently enrolled in care and of these, 778 (11.3%) self-identified as MSM. A cross-sectional study which used a convenience sample was conducted over the period September 2020 –June 2021, where all MSM 18 years and over attending the clinic received information about the study protocol and procedures and were asked to participate in the study after written informed consent. To protect patient confidentiality, each study participant was allocated a unique identifying number and a structured questionnaire was administered that collected socio-demographic information, alcohol and drug use, history of STIs, access to health care and sexual behaviours. A chart review was conducted to obtain data on the syphilis serology and HIV viral loads in the study patients.

### Inclusion criteria

1. Persons living with HIV
2. Patients 18 years and older
3. Patients who attend the MRFTT for HIV treatment and care
4. Self-identified as gay, bisexual or other MSM

### Exclusion criteria

1. Patients under 18 years of age
2. Female sex
3. HIV seronegative individuals
4. Heterosexuals

To calculate the sample size at the 95% confidence level with a margin of error (confidence interval) of 5, using a population of size of 778 MSM at a population proportion of 50%, the calculated sample size would be 258 study patients.

### Ethical approval

The study protocol, data collecting instruments and informed consent form were approved by the Campus Research Ethics Committee of the University of the West Indies, St. Augustine, Trinidad, approval number CREC-SA.0489/08/2020.

### Testing for syphilis at the MRFTT

At the HIV Clinic, testing for syphilis in MSM was conducted once a year (or as required by the treating physician) at the MRFTT Laboratory using the traditional screening algorithm for the diagnosis of syphilis. Here, sera were screened with a quantitative nontreponemal assay, the Venereal Disease Research Laboratory (VDRL) test (Becton, Dickinson and Company, Franklin Lakes, NJ, USA) with confirmation of the reactive samples using a treponemal assay, the Treponema Pallidum Particle Agglutination (TPPA) test (Serodia TPPA test kit, Fujireibo Inc., Tokyo, Japan). To assess, monitor and improve the reliability and accuracy of the diagnostic tests, standardization and external quality proficiency assessments were performed quarterly through Oneworld Accuracy, Canada [13].

Using the traditional algorithm, a diagnosis of syphilis was made by a reactive VDRL test confirmed by a reactive TPPA. A confirmed serological test result for syphilis is indicative of the presence of treponemal antibodies but may be unable to distinguish between past and current infection, thus correlation from the history of the patient ever having a diagnosis of syphilis and subsequent treatment is important [11] and this was obtained by chart review.

A syphilis diagnosis (active syphilis) was defined in the study as a patient with:

1. a reactive VDRL test confirmed by a reactive TPPA in a patient with no previous reactive syphilis serology or no history of syphilis or no previous treatment for syphilis
2. a reactive VDRL and TPPA serology following a diagnosis of syphilis and after treatment there was a subsequent ≥4-fold reduction or negativity of the VDRL titres followed by a consecutive ≥4-fold titre increase with a titre of at least 8 in VDRL testing [11] suggesting syphilis re-infection.

A previous syphilis diagnosis was defined in the study as a patient with representing

1. Recently treated syphilis with VDRL titres that have not yet become non-reactive [14]
2. Adequately treated syphilis with persistent titres [14]. The patient had a reactive VDRL and TPPA serology following a diagnosis of syphilis and after treatment there was a subsequent ≥4-fold reduction of the VDRL titres but persistent VDRL titres remained without seroreversion.

Patients were considered as symptomatic if they were diagnosed with primary syphilis or secondary syphilis and asymptomatic if diagnosed with latent syphilis (early or late latent syphilis/syphilis of unknown duration).

### Data collection and data analysis

Responses collected from the structured questionnaire were entered into an excel format, cleaned and coded into numerical variables. These numeric variables were then transferred to an IBM Statistical Package for Social Science (SPSS) Version 25 format for statistical analysis. New variables were created as a result of the responses obtained including, syphilis diagnosis, previously diagnosed syphilis, alcohol use, marijuana consumption in the last 12 months, cocaine consumptions in the last 12 months, sex under the influence of alcohol, sex under the influence of drugs, group sex, anonymous sex, condom use with anonymous sex, condom use with partner, disclosure to partner, disclosure to anonymous sex partner, receptive anal sex without a condom. All of these variables were dichotomous in nature.

Numeric variables were summarized using descriptive statistics such as frequencies, the mean, median and standard deviation. Chi-square tests (X^2^) and other bivariate analysis such as t-test were conducted to examine associations between the independent variables and the diagnosis of syphilis and those with a previous syphilis diagnosis. This analysis was used where appropriate to identify if a statistically significant association in the nominal variables ‘Syphilis Diagnosis’ and ‘Previous Syphilis Diagnosis’ by ethnicity, income, employment status, sexual orientation (homosexual and bisexual activity), the clinical status (symptomatic vs asymptomatic) and the dichotomous variables listed above.

To identify the independent predictors of patients diagnosed with syphilis and previously diagnosed with syphilis, multivariable logistic regression analyses were conducted. The independent variables included all the variables that were statistically significant in the bi-variate analyses. The results were presented as odds ratios (95% CI).

## Results

During the study period, 264 MSM living with HIV were screened for syphilis, age range 18–64 years, median age 33 years and 34.4% were actively bisexual, 93.4% of the patients were on ART and 82.2% had an undetectable viral load (viral load < 40 copies/ml) [Table 1].

**Table 1:** Baseline characteristics of the study population (n = 264).

Descriptive Statistic	Total (n = 264)
**Median Age**	33.0 years
**Age Range**	18–64 years
**Age group (years)**	
**<24**	34 (12.9%)
**25–29**	53 (20.1%)
**30–34**	60 (22.7%)
**35–39**	41 (15.5%
**40–49**	37 (14%)
**50+**	39 (14.8%)
**Ethnicity (n = 261)**	
African	122 (46.7%)
East Indian	35 (13.4%)
Mixed/Other	104 (39.8%)
**Sexual Orientation (n = 259)**	
Gay/Homosexual	170(65.6%)
Bisexual	89 (34.4%)
**Married or Common Law with Man (n = 261)**	
Yes	65 (24.9%)
No	196 (75.1%)
Patient on ART	247 (93.4%)
Undetectable Viral load [Viral load<40 copies/ml]	217 (82.2%)

In the study, 28% (74/264) of patients had a diagnosis of syphilis and of these 2 (2.7%) were diagnosed with primary syphilis, 6 (8.1%) were diagnosed with secondary syphilis, 24 (32.4%) early latent syphilis (ELS), 42 (56.8%) with late latent syphilis (LLS)/syphilis of unknown duration. Therefore 10.8% (8/74) of patients were symptomatic (primary and secondary syphilis) and 89.2% (66/74) were asymptomatic (ELS and LLS).

On screening for syphilis, 122 study patients had positive syphilis serology, of whom 39.3% (48/122) patients had a previous diagnosis of syphilis with residual reactive VDRL serology (with titres ranging from 1:2 to 1:32) and TPPA tests after adequate treatment, representing recently treated syphilis with VDRL titres that had not yet become non-reactive or patients with persistent titres. These patients were therefore not included as cases of syphilis in this study.

Table 2 shows the statistically significant correlates of prevalent syphilis in MSM living with HIV who were diagnosed with syphilis including those who consumed more than 6 drinks of alcohol in one sitting multiple times (p = 0.021), those who used marijuana in the past 12 months (p = 0.019), those with a history of a previous STI (p = 0.014), those with a previous diagnosis of syphilis (0.002), those who previously sought treatment for syphilis (p = 0.043), those who had sex with anonymous partners (p = 0.034), those who did not use a condom with anonymous sex (p = 0.015) and those who disclosed their HIV status to partners (p = 0.023). There were no statistically significant associations among the other correlates of prevalent syphilis and a positive diagnosis of syphilis including condomless anal sex, meeting partners on sexual networking sites on the internet, serosorting and other high risk behaviours in the bivariate analysis.

**Table 2:** Statistically significant correlates of prevalent syphilis in MSM living with HIV with a diagnosis of syphilis.

Variable	Total number of patients n = 264 (%)	Positive Diagnosis with syphilis n = 74 (%)	P value	OR	95% CI
**Consumed 6 or more Drinks (n = 259)**					
Zero	118 (45.6%)	35 (47.9%)	0.021	Reference	1.24–166.22
1 to 3 times	97 (37.5%)	18 (24.7%)			
4 to 6 times	27 (10.4%)	13 (17.8%)			
7 to 9 times	6 (2.3%)	2 (2.7%)			
More than 10 times	11 (4.2%)	5 (6.8%)		14.35	
Marijuana use in the last 12 months (n = 259)					
Yes	73 (28.2%)	42 (57.5%)	0.019	1.92	1.11–3.32
No	186 (71.8%)	31 (42.5%)			
Had a previous STI (n = 249)					
Yes	63 (25.3%)	25 (36.2%)	0.014	2.12	1.16–3.90
No	186 (74.7%)	44 (63.8%)			
Previous diagnosis of Syphilis (n = 172)					
Yes	57 (33.1%)	25 (51.0%)	0.002	2.96	1.49–5.91
No	115 (66.9%)	24 (49.0%)			
Sought Treatment for Syphilis (n = 75)					
Yes	37 (49.3%)	17 (65.4%)	0.043	2.74	1.02–7.36
No	38 (50.7%)	9 (34.6%)			
Sex with anonymous partners (n = 244)					
Yes	53 (21.5%)	21 (30.4%)	0.034	1.98	1.05–3.76
No	193 (78.5%)	48 (69.6)			
No condom Use with Anonymous Sex (n = 119)					
Yes	90 (75.6%)	33 (91.7%)	0.010	5.02	1.41–17.86
No	29 (24.4%)	3 (8.3%)			
Disclosure (told sex partner HIV status) [n = 244]					
Yes	161 (66.0%)	51 (77.3%)	0.023	2.10	1.10–4.03
No	83 (34.0%)	15 (22.7%)			

Multivariable logistic regression analysis of the independent factors [Table 3] showed that having a diagnosis of syphilis was more common in those patients who used marijuana in the last 12 months (OR 2.13; 95% CI, 1.14–3.96), participated in anonymous sex (OR 2.09; 95% CI, 1.03–4.26), those who disclosed their HIV status to their sex partner (OR 2.23; 95% CI, 1.11–4.49) and those with a previous diagnosis of syphilis (OR 5.16; 95% CI, 1.03–25.83).

**Table 3:** Results of multivariable logistic regression of the independent correlates of prevalent syphilis for having a diagnosis of syphilis among MSM living with HIV.

Variable	OR	95% CI	p value
Marijuana use in the last 12 months	2.13	1.14–3.96	0.017
Sex with anonymous partners	2.09	1.03–4.26	0.042
Disclosure (told sex partner HIV status)	2.23	1.11–4.49	0.025
Previous diagnosis of syphilis	5.16	1.03–25.83	0.046

## Discussion

In this study, the prevalence of syphilis in MSM living with HIV was 28% and MSM with a previous diagnosis of syphilis were five times more likely to be diagnosed with syphilis (OR 5.16; 95% CI, 1.03–25.83). These findings are similar to a retrospective analysis in MSM living with HIV conducted at the MRFTT in 2019 which found a high prevalence of syphilis of 41.3% [11] and patients with a previous history of treated syphilis were ten times more likely to be diagnosed with syphilis [11]. Similar results were demonstrated by Roth et al in a study of a Swiss cohort of MSM living with HIV where repeat episodes of syphilis were reported [15] and this was attributed to high risk sexual behavior and the high background rate of syphilis in MSM populations [15]. Thus, in MSM living with HIV in Trinidad, repeat episodes of syphilis may to play a role in the transmission dynamics of *Treponema pallidum* infection in this group of patients who may be core transmitters of syphilis [16].

In our study, most of the syphilis infections were asymptomatic (89.2%). This was also reported in the retrospective study of MSM attending the HIV Clinic in Trinidad in 2019 with 71.1% of these infections being asymptomatic [11] and by Branger et al [17] in an outpatient clinic in Amsterdam where routine syphilis screening conducted on 1,105 HIV infected patients identified 81 cases with syphilis, all among males of whom 94% were MSM with 33% of the infections being asymptomatic. A few studies have suggested that persons with past episodes of syphilis were more likely to be asymptomatic on repeat episodes [18,19] possibly due to the acquired immune response resulting from past infection which may attenuate the clinical manifestations of reinfection with *T*. *pallidum* [19]. In our study, some of the MSM stated that since they were HIV infected, oral sex was used as a “safer sexual method” to reduce transmission of HIV, however studies have shown that there was a strong association between syphilis infection and unprotected oral sex [20,21].

It was found that those who disclosed their HIV status to their sex partners were twice as likely to be diagnosed with syphilis (OR 2.23; 95% CI, 1.11–4.49) which is a marker of unprotected sex. This is supported in a study by Cook et al in Florida [22] where it was found that MSM who disclosed their HIV status to sexual partners were three times more likely to participate in condomless sex [22]. These findings are not consistent with data from most other studies which showed that MSM adopt safer sexual practices following disclosure [23–26]. Disclosure of HIV status to their sexual partners by MSM may improve their psychological well-being as this may result in improved emotional support which may be associated with reduced anxiety and depression [27], on the other hand disclosure of their HIV status may result in rejection by the sexual partner, stigma and isolation [27]. Nondisclosure to a sexual partner has been linked with nonacceptance of one’s HIV positivity and a desire for this information to be kept private [28].

Multivariable logistic regression analysis showed that having a diagnosis of syphilis was more common in those patients who participated in anonymous sex (OR 2.09; 95% CI, 1.03–4.26). It was reported in King County Washington that MSM who practiced anonymous sex with multiple partners were the primary drivers for the reintroduction of syphilis among MSM [29]. It was also shown that patients diagnosed with syphilis in our study were more likely to have used marijuana in the last 12 months (OR 2.13; 95% CI, 1.14–3.96). A systematic review conducted by Tomkins et al [30] reviewed the evidence of the relationship between sexual activity among MSM and recreational drug use and found links between high risk sexual behaviours and sexualized drug taking in several studies which were associated with the acquisition of STIs including syphilis [30] with the most commonly used drugs being metamphetamines, ectasy, cocaine, erectile dysfunction drugs and cannabis/marijuana (30). Conversely, a study reported that exclusive cannabis use [30] among MSM was associated with fewer STIs and a reduced number of sexual encounters [31] and suggested that cannabis may not necessarily increase sexual desire or stimulation [31].

On completion of the retrospective study [11], a pilot intervention was put in place at the MRFTT in October 2020 to reduce the transmission of syphilis in this population in the form of a once weekly men’s health clinic with a telemedicine component where all the MSM with a history of positive syphilis serology were referred to the clinic. Activities in the pilot intervention included six monthly screening and treatment of STIs, partner notification and treatment strategies, education on safe sex practices and risk reduction, adherence to ART and viral suppression, condom distribution and vaccination for HPV and hepatitis B. This pilot intervention, although only in place for a few months, may have contributed in part to the prevalence of syphilis among MSM living with HIV being reduced from 41.3% in 2019 to 28% in this study. However, the COVID-19 pandemic lockdown measures may have decreased the opportunities for sexual activities in MSM and may have reduced the likelihood of access for diagnosis and treatment of syphilis and other STIs [3]. Missed opportunities with syphilis screening may have resulted in an underestimation in the numbers of MSM diagnosed with syphilis in this study.

Of the 6,883 patients currently enrolled in care at the HIV Clinic in Trinidad, 778 (11.3%) self-identified as MSM which is probably an underestimate as some patients may not feel comfortable disclosing their sexual orientation to members of the clinical team. In addition, some patients may be married and do not want their wives/family finding out about their sexual activities and some men self-identify as heterosexual even though they may have sex with men [32].

Combination prevention strategies are needed in MSM living with HIV inclusive of comprehensive clinical services involving the routine screening for syphilis and other STIs, diagnosis and treatment which is an important initiative as many of these infections are asymptomatic and early identification of syphilis and treatment will shorten the duration of infectivity and assist in controlling the epidemic. In addition, targeted prevention methods are important including behavioural risk reduction strategies, increased condom use, partner notification and treatment to interrupt the chain of transmission, substance abuse prevention programmes, pre-exposure vaccination (HPV and hepatitis B) and adherence to ART to maintain viral suppression and reduce the transmission of HIV.

### Study limitations

Our study has a number of limitations as there is stigma and discrimination associated with being MSM so some patients may not have felt comfortable disclosing their sexual orientation thereby resulting in imprecision and an underestimate of the population of MSM living with HIV attending the clinic. This was a cross-sectional study design with convenience sampling of self-identified MSM attending the HIV clinic so the results may not be generalizable to HIV infected population of MSM living in T&T. Limiting the study to MSM living with HIV may not be representative of the population of MSM (some of whom may be HIV uninfected) and may have biased the study results. During the COVID-19 lockdown measures, missed opportunities with syphilis screening may have resulted in an underestimation in the numbers of MSM diagnosed with syphilis in this study. Even though the self-administered questionnaire had no names or identifiers on it, some patients may not have felt comfortable disclosing their true sexual behaviours for fear of stigma or reproach by health care providers and this may result in bias and inaccuracy of data collected.

## Conclusion

There is a high prevalence of asymptomatic syphilis among MSM who attend the HIV clinic in Trinidad. The patients more likely to be diagnosed with syphilis were those who participated in sex with anonymous partners (OR 2.09; 95% CI, 1.03–4.26), those with a previous diagnosis of syphilis (OR 5.16; 95% CI, 1.03–25.83), those who used marijuana in the last 12 months (OR 2.13; 95% CI, 1.14–3.96) and those who disclosed their HIV status to their sex partner (OR 2.23; 95% CI, 1.11–4.49). Repeat episodes of syphilis and sex with anonymous partners seem to play a role in the transmission dynamics of *T pallidum* infection among MSM living with HIV and a pilot intervention put in place in October 2020 may have resulted in a reduction in the prevalence of syphilis in this population. Thus combination prevention approaches including the expansion of the pilot intervention are urgently needed to reduce syphilis transmission.

## Supporting information

S1 File Syphilis in MSM behavioural questionnaire PLOS. (DOCX)

S2 File Syphilis in MSM behavioural database June 2021 PLOS. (XLSX)
