# Supplementary material for: Correlates of prevalent syphilis infection among men who have sex with men (MSM) living with HIV attending the HIV clinic in Trinidad
Source: PLoS One. 2022 Mar 31;17(3):e0265909. doi: 10.1371/journal.pone.0265909 (PMC8970382; doi:10.1371/journal.pone.0265909)
Supplement: S1 File — (DOCX) [file pone.0265909.s001.docx]

**QUESTIONNAIRE**

Study ID Number ……………………… Sex Male

HIV Status Known Newly Diagnosed

Age :

**Section 1: Socio Demographics**

1. How old were you at your last birthday? __ __ __

1. What is the highest level of school that you have ever attained? (Choose one)

01 None

02 Primary

03 Secondary

04 Vocational

05 Tertiary/University

1. Please tell me which choice best describes your employment status (Choose one)

01 Employed

02 Self Employed

03 Unemployed

1. What is your nationality? (Choose one)

01 Trinidad and Tobago

02 Jamaica

03 Barbados

04 Guyana

05 St Vincent

06 Grenada

07 Other (please specify below)

If question 4 is equal to 07, then please tell us what your nationality is

__ __ __ __ __ __ __ __ __ __ __ __ __ __ __ __ __ __ __ __ __ __ __ __ __ __

1. Which area do you currently live in? (Choose one)

01 City of Port of Spain

02 Borough of Chaguanas

03 Arima and Environs

04 San Juan/Laventille

05 Mayaro/Rio Claro

06 Penal/Debe/Siparia

07 Borough of Point Fortin

08 City of San Fernando

09 Diego Martin/Petit Valley/Carenage

10 Tunapuna/Piarco

11 Borough of Arima

12 Sangre Grande

13 Couva/Tabaquite/Talparo

14 Princes Town

15 Tobago

16 Other

17 Outside Trinidad and Tobago

1. Which ethnic group do you belong to? (Choose one)

01 African Origin

02 Caucasian

03 Chinese

04 East Indian Origin

05 Mixed

06 Syrian/Lebanese

07 Other Ethnic Group (please specify below)

If question 6 is equal to 07, then please tell us what your ethnicity is

__ ___ ___ ____ ____ ____ ____ ____ ____ ____ ____ ____ ____ ____

1. Are you **currently** married or living in a common law relationship with a woman or man ?
   - - 1. No
       2. Yes, with a man
       3. Yes, with a woman

1. What is currently your **main** source of income? (Choose one)

01 Employment income (including self-employment income)

State main occupation (e.g.: secondary school teacher, accounts clerk, automobile mechanic):_______________________________________

02 No Income

03 Income received as a sex worker

04 Income from rent

05 Pensions and Annuities.

06 National Insurance Benefits

07 Old age pensions

08 Public assistance

09 Financial assistance from sexual partners

10 Financial assistance from person who are not sexual partners (ex: friends, household members, family, etc.)

11 Other income (please specify below)

If question 8 is equal to 11, then Please tell us your main source of income.

__ __ __ __ __ __ __ __ __ __ __ __ __ __ __ __ __ __ __ __ __ __ __ __ __ __

1. What was your average total gross income **per month** (in Trinidad and Tobago
    currency) over the last 12 months? (Choose one)

01 None

02 <$500

03 $500 - $2999 per month

04 $3000 - $5999 per month

06 $6000 - $8999 per month

07 > $9000 per month

**Section 2: Alcohol and Drug Use**

1. In the last 30 days, how many times did you consume six (6) or more drinks
    containing alcohol in one sitting?

01 zero (0)

02 1 to 3

03 4 to 6

04 7 to 9

05 More than 10

1. In the last 30 days, on how many days did you have sex while under the influence of alcohol? __ _____

01 zero (0)

02 1 to 3

03 4 to 6

04 7 to 9

05 More than 10

1. Some people use different types of ‘street’ drugs. In the last 12 months, which of the following, if any, have you used?(Check all that apply)

01 Marijuana/weed

02 Cocaine- crack

03 Cocaine- powder

04 Heroin

05 Ecstasy

06 Fantasy

07 Other (please specify below)

08 I have not tried any of the above

***If question 12 is equal to 07,*** *then what other types of non-injection drugs have you used?*

__ __ __ __ __ __ __ __ __ __ __ __ __ __ __ __ __ __ __ __ __ __ __ __ __ __

1. Have you ever shared needles for “shooting up” or injected any drugs into your body using shared needles, not including ones prescribed for you?

01 Yes

00 No

1. In the **last 30 days**, on how many days have you had sex while under the influence of any drug you had used?__ ___

01 zero (0)

02 1 to 3

03 4 to 6

04 7 to 9

05 More than 10

**Section 3: Healthcare Access and STIs**

1. Have you had symptoms of a sexually transmitted infection in the last 12 months?

01 Yes

00 No

1. Have you been told that you have the following sexually transmitted infections in the last 12 months, and if so, have you been treated?

|  | **Been told that you have** | | **Sought treatment** | |
| --- | --- | --- | --- | --- |
| Syphilis | Yes_01_ | No_00_ | Yes_01_ | No_00_ |
| Herpes | Yes_01_ | No_00_ | Yes_01_ | No_00_ |
| Genital warts | Yes_01_ | No_00_ | Yes_01_ | No_00_ |
| Gonorrhea | Yes_01_ | No_00_ | Yes_01_ | No_00_ |
| Chlamydia | Yes_01_ | No_00_ | Yes_01_ | No_00_ |
| HIV | Yes_01_ | No_00_ | Yes_01_ | No_00_ |
| Other | Yes_01_ | No_00_ | Yes_01_ | No_00_ |

1. Did you notify your last sexual partner of your sexually transmitted disease?

01 Yes

00 No Skip to next question

98 Don't Know Skip to next question

97 Not Applicable Skip to next question

1. Are any of the following reasons why you did not notify your last sexual partner of
    your sexually transmitted disease treatment? (Check all that apply)

01 I was afraid partner would be upset

02 I was afraid partner might be violent against me

03 I did not know partner/how to locate partner

04 I did not feel it was necessary to discuss with partner

05 I was too embarrassed to discuss with partner

06 Other

97 Not Applicable

If question 18 is equal to 06, then what are the other reason(s) you chose not to notify your last sexual partner of your sexually transmitted disease treatment.

__ __ __ __ __ __ __ __ __ __ __ __ __ __ __ __ __ __ __ __ __ __ __ __ __ __ __ __

**Section 4: Sexual Behaviors**

1. Have you ever had oral, vaginal or anal sex in the past 12 months? __ __

01 Yes

00 No **Skip to Q25**

1. In total, with how many different partners have you had oral, vaginal or anal sex in
    the past 12 months __ __

01 I haven’t had sex within the last 12 months

02 1 to 3

03 4 to 6

04 7 to 9

05 More than 10

1. Of those sexual partners in the last 12 months, for how many do you know their HIV
    status? __ __

98 Don't Know

1. In the last 12 months, when you had sex with your partners, how often do you use a condom?

01 Always

02 Sometimes

03 Never

1. Of all your sexual partners in the past 12 months, how many were men or women **you gave** money, goods or services to have sex with you? __ __

98 Don't Know

1. Of all your sexual in the last 12 months, how many were men or women who **gave you** money, goods or services in exchange for sexual acts?__ __

98 Don't Know

99 Refuse to Answer

**Now we would like to ask you about your most recent sexual partner.**

1. The last time you had sex with your **most recent partner** did you and your partner use a condom?

01 Yes **Skip to question 27**

00 No

1. Why did you not use a condom? (Check all that apply)

01 Partner refused

02 I refused

03 I trust/know my partner

04 My partner and I had the same HIV status

05 Condom was not available

06 I was under the influence of alcohol or drugs

07 I was afraid of violence/threat from partner

08 Sex feels better without a condom

09 Condoms are too expensive

10 I was paid extra not to use a condom

88 Other (please specify below)

98 Don't Know

If Question 26 is equal to 88, then what is the other reason that you did not use a condom?

__ __ __ __ __ __ __ __ __ __ __ __ __ __ __ __ __ __ __ __ __ __ __ __ __ __ __ __

1. In the past 12 months, did you participate in receptive anal sex without using a condom?

00 Yes

01 No

1. In the last 12 months, when you had receptive anal sex with your partners, how often do you use a condom?
2. Always
3. Sometimes
4. Never

29. In the past 12 months, did you participate in sex with more than one person at a time (group sex)?

01 Yes

00 No

98 Don't Know

30. In the last 12 months, when you had group sex, how often did you use a condom?

01 Always

02 Sometimes

03 Never

31. In the past 12 months, did you participate in sex with persons you never met before (anonymous sex)?

01 Yes

00 No

98 Don't Know

32. In the last 12 months, when you had anonymous sex, how often did you use a condom?

01 Always

02 Sometimes

03 Never

33. In the last 12 months, when you had anonymous sex, where did you meet your new partner?

01 At a social event/party

02 Introduced via a friend

03 On the internet/social media

04 At work

05 Other

34. Do you know the HIV status of your most recent sexual partner?

01 Yes – HIV positive

02 Yes – HIV negative

1. Don't Know

35. If you are HIV positive, do you only have sex with persons who are HIV positive (serosorting)?

01 Yes

00 No

1. Don't Know
2. Did you tell your most recent sex partner your HIV status?

01 Yes

00 No

98 Don't Know

37. If you are HIV positive, are you on medication to treat HIV (HAART)?

01 Yes

00 No

1. Don't Know

38. What is your most recent HIV viral load?

01 Undetectable

00 Between 20-1000 copies/ml

98 >1000 copies/ml

98 Don't Know

**Section 5: Sexual Identity**

1. Which of the following terms best describes your sexual identity? (Choose one)

01 Gay

02 Homosexual

03 Bisexual

04 Transsexual

05 Heterosexual

06 Drag Queen

07 Bisexual

08 Transgendered

88 Other (please specify below)

98 Don't Know

If question 39 is equal to 88, then please tell us what word you would use to describe your sexual identity

__ __ __ __ __ __ __ __ __ __ __ __ __ __ __ __ __ __ __ __ __ __ __ __ __ __

1. Which of the following best describes who you are attracted to? (Choose one)

01 Men

02 Women

03 Mix, more men

04 Mix, more women

05 Even mix

06 Neither

99 Refuse to Answer
